# Chromosome-scale assembly of the Verbenaceae species Queen’s Wreath (*Petrea volubilis L.*)

**DOI:** 10.1186/s12863-023-01110-z

**Published:** 2023-03-03

**Authors:** John P. Hamilton, Brieanne Vaillancourt, Joshua C. Wood, C. Robin Buell

**Affiliations:** 1 Center for Applied Genetic Technologies, University of Georgia, Athens, GA 30602 USA; 2 Department of Crop & Soil Sciences, University of Georgia, Athens, GA 30602 USA; 3 Institute of Plant Breeding, Genetics, & Genomics, University of Georgia, Athens, GA 30602 USA

**Keywords:** Queen's wreath, Genome assembly, Lamiales, Comparative genomics, Petrea volubilis

## Abstract

**Objectives:**

*Petrea volubilis*, a member of the Order Lamiales and the Verbenaceae family, is an important horticultural species that has been used in traditional folk medicine. To provide a genome sequence for comparative studies within the Order Lamiales that includes important families such as Lamiaceae (mints), we generated a long-read, chromosome-scale genome assembly of this species.

**Data description:**

Using a total of 45.5 Gb of Pacific Biosciences long read sequence, we generated a 480.2 Mb assembly of *P. volubilis,* of which, 93% is chromosome anchored. Representation of genic regions was robust with 96.6% of the Benchmarking of Universal Single Copy Orthologs present in the genome assembly. A total of 57.8% of the genome was annotated as a repetitive sequence. Using a gene annotation pipeline that included refinement of gene models using transcript evidence, 30,982 high confidence genes were annotated. Access to the *P. volubilis* genome will facilitate evolutionary studies in the Lamiales, a key order of Asterids that includes significant crop and medicinal plant species.

**Supplementary Information:**

The online version contains supplementary material available at 10.1186/s12863-023-01110-z.

## Objective

The Asterid species, *Petrea volubilis* L., also known as Queen’s Wreath, Purple Wreath, Bluebird vine or Sandpiper vine, is a member of the Verbenaceae family within the Order Lamiales. As a perennial woody vine, *P. volubilis* is a key ornamental species due to its intense violet flowers. Historically, leaves of *P. volubilis* have been used in Mexico as folk medicine to remedy kidney stones, rheumatism, diarrhea, and urinary infections [1] and as an abortifacient in Jamaica [2]. *P. volubilis* extracts have been found to have antipyretic, analgesic, and anti-microbial [3, 4] and insecticidal activities [4]. Recently, *P. volubilis* was included as one of four outgroup species in a study that revealed the evolutionary basis of chemical diversity in the Lamiacaeae [5]. In this project, we sequenced and annotated the *P. volubilis* genome to facilitate our understanding of genome and chemodiversity evolution within the Lamiales.

## Data description

High molecular weight DNA was isolated using a modified cetyl trimethylammonium bromide method (2% CTAB, 100 mM Tris, 1.4 M Sodium Chloride, 20 mM EDTA) [6] followed by RNase treatment and cleanup using the DNeasy PowerClean Pro Cleanup Kit (Qiagen). Pacific Biosciences (PacBio) SMRTbell Express Template libraries were constructed and sequenced on a PacBio Sequel instrument generating 45.5 Gb of total sequence (Table 1, Data file 1, Data sets 1 & 2, [7]). Reads less than 5 kb were filtered out and the remaining reads were assembled using Canu v1.8 [8] with the options: minOverlapLength = 2000 minReadLength = 5000 genomeSize = 450 m resulting in an initial assembly of 630.0 Mb with 6,515 contigs and an N50 contig length of 369,179 bp. The genome was polished with two rounds of GCpp (v1.9.0) [9], followed by three rounds of polishing with Pilon (v1.23) [10] using Illumina whole genome shotgun reads (Table 1, Data file 1, Data set 3, [7, 11]). A k-mer distribution plot using GenomeScope [12] revealed the genome was heterozygous (Table 1, Data file 2, Data set 3, [7]). Haplotigs were removed using two rounds of purge_dups using the default parameters (v1.0.0) [13, 14] and Hi-C libraries constructed by Phase Genomics (Table 1, Data file 1, Data sets 4 & 5, [7, 15, 16]) were used to place the final scaffolds into 17 chromosomes using the Juicer (v1.6)/3D-DNA pipeline (git commit: 529ccf4; Table 1, Data file 3) [7, 17, 18]. The final assembly size is 480.2 Mb (478.8 Mb ungapped, 93% chromosome-anchored), consistent with the size estimated by flow cytometry of 455 Mb per 1C [5] (Table 1, Data files 4 & 5, [7]). A comparison of k-mers in the Illumina whole genome shotgun reads vs the genome assembly using KAT (v2.4.1) [19] with a k-mer size of 21 revealed that *P. volubilis* is heterozygous (estimated heterozygosity rate 1.45%) and the assembly is near-complete (estimated completeness, 98.8%;(Table 1, Data file 6, [7]). The majority of k-mers in the reads are present in one copy indicating the haplotigs were successfully purged from the final assembly (Table 1, Data files 1 & 6, Data set 3, [7]). Assessment of representation of genic regions using the Benchmarking of Universal Single Copy Orthologs [20] (BUSCO; v5.4.3 with embryophyta_odb10) revealed 96.6% of the BUSCO genes present in the genome assembly (Table 1, Data file 7, [7]). While the scaffold N50 was 25.6 Mb, the contig N50 was 0.53 Mb due potentially to heterozygosity that reduced the ability of the assembler to generate longer contigs (Table 1, Data file 6, [7]; see Limitations).

**Table 1:** Overview of data files and data sets used in this study

Label	Data file/Data set name	File types	Data repository and identifier (DOI or accession number)
Data file 1	*Petrea volubilis* libraries used in this study	Spreadsheet (.xlxs)	Figshare (https://doi.org/10.6084/m9.figshare.21429219.v3) [7]
Data file 2	Genomescope k-mer frequency distribution plot	Portable Document Files (.pdf)	Figshare (https://doi.org/10.6084/m9.figshare.21429219.v3) [7]
Data file 3	Hi-C contact map	Portable Document Files (.pdf)	Figshare (https://doi.org/10.6084/m9.figshare.21429219.v3) [7]
Data file 4	Assembly metrics for the *Petrea volubilis* assembly	Spreadsheet (.xlxs)	Figshare (https://doi.org/10.6084/m9.figshare.21429219.v3) [7]
Data file 5	Pseudomolecule lengths and gap content for the *Petrea volubulis* assembly	Spreadsheet (.xlxs)	Figshare (https://doi.org/10.6084/m9.figshare.21429219.v3) [7]
Data file 6	KAT k-mer comparison plot	Portable Document Files (.pdf)	Figshare (https://doi.org/10.6084/m9.figshare.21429219.v3) [7]
Data file 7	BUSCO results on the *Petrea volubilis assembly* and annotation	Spreadsheet (.xlxs)	Figshare (https://doi.org/10.6084/m9.figshare.21429219.v3) [7]
Data file 8	Repetitive sequence content in the *Petrea volubilis* assembly	Spreadsheet (.xlxs)	Figshare (https://doi.org/10.6084/m9.figshare.21429219.v3) [7]
Data file 9	*Petrea volubulis* gene annotation summary	Spreadsheet (.xlxs)	Figshare (https://doi.org/10.6084/m9.figshare.21429219.v3) [7]
Data set 1	Pac Bio reads from high molecular weight DNA, SRR11516643	Fastq file (.fastq.gz)	NCBI (https://identifiers.org/ncbi/insdc.sra:SRR11516643) [7, 21]
Data set 2	Pac Bio reads from high molecular weight DNA, SRR11516644	Fastq file (.fastq.gz)	NCBI (https://identifiers.org/ncbi/insdc.sra:SRR11516644) [7, 22]
Data set 3	Illumina whole genome shotgun reads, SRR11516645	Fastq file (.fastq.gz)	NCBI (https://identifiers.org/ncbi/insdc.sra:SRR11516645) [7, 11]
Data set 4	Illumina Hi-C DNA sequence reads, SRR15904679	Fastq file (.fastq.gz)	NCBI (https://identifiers.org/ncbi/insdc.sra:SRR15904679) [7, 15]
Data set 5	Illumina Hi-C DNA sequence reads, SRR15904680	Fastq file (.fastq.gz)	NCBI (https://identifiers.org/ncbi/insdc.sra:SRR15904680) [7, 16]
Data set 6	Illumina RNA-Seq—Root, SRR8937863	Fastq file (.fastq.gz)	NCBI (https://identifiers.org/ncbi/insdc.sra:SRR8937863) [7, 23]
Data set 7	Illumina RNA-Seq—Petiole, SRR8937861	Fastq file (.fastq.gz)	NCBI (https://identifiers.org/ncbi/insdc.sra:SRR8937861) [7, 24]
Data set 8	Illumina RNA-Seq—Stem, SRR8937862	Fastq file (.fastq.gz)	NCBI (https://identifiers.org/ncbi/insdc.sra:SRR8937862) [7, 25]
Data set 9	Illumina RNA-Seq—Immature leaf, SRR8937859	Fastq file (.fastq.gz)	NCBI (https://identifiers.org/ncbi/insdc.sra:SRR8937859) [7, 26]
Data set 10	Illumina RNA-Seq—Mature leaf, SRR8937860	Fastq file (.fastq.gz)	NCBI (https://identifiers.org/ncbi/insdc.sra:SRR8937860) [7, 27]
Data set 11	Genome assembly of *Petrea volubilis*	fasta file (.fa)	NCBI (https://identifiers.org/assembly:GCA_026212405.1) [7, 28]
Data set 12	High Confidence *Petrea volubilis* Gene Models cDNA	fasta file (.fa)	Figshare (https://doi.org/10.6084/m9.figshare.21429219.v3) [7]
Data set 13	High Confidence *Petrea volubilis* Gene Models CDS	fasta file (.fa)	Figshare (https://doi.org/10.6084/m9.figshare.21429219.v3) [7]
Data set 14	High Confidence *Petrea volubilis* Gene Models GFF3	GFF3 file (.gff3)	Figshare (https://doi.org/10.6084/m9.figshare.21429219.v3) [7]
Data set 15	High Confidence *Petrea volubilis* Gene Models Proteins	fasta file (.fa)	Figshare (https://doi.org/10.6084/m9.figshare.21429219.v3) [7]
Data set 16	Representative High Confidence *Petrea volubilis* Gene Models cDNA	fasta file (.fa)	Figshare (https://doi.org/10.6084/m9.figshare.21429219.v3) [7]
Data set 17	Representative High Confidence *Petrea volubilis* Gene Models CDS	fasta file (.fa)	Figshare (https://doi.org/10.6084/m9.figshare.21429219.v3) [7]
Data set 18	Representative High Confidence *Petrea volubilis* Gene Models GFF3	GFF3 file (.gff3)	Figshare (https://doi.org/10.6084/m9.figshare.21429219.v3) [7]
Data set 19	Representative High Confidence *Petrea volubilis* Gene Models List	text file (.txt)	Figshare (https://doi.org/10.6084/m9.figshare.21429219.v3) [7]
Data set 20	Representative High Confidence *Petrea volubilis* Gene Models Proteins	fasta file (.fa)	Figshare (https://doi.org/10.6084/m9.figshare.21429219.v3) [7]
Data set 21	*Petrea volubilis* Working Gene Models cDNA	fasta file (.fa)	Figshare (https://doi.org/10.6084/m9.figshare.21429219.v3) [7]
Data set 22	*Petrea volubilis* Working Gene Models CDS	fasta file (.fa)	Figshare (https://doi.org/10.6084/m9.figshare.21429219.v3) [7]
Data set 23	*Petrea volubilis* Working Gene Models GFF3	GFF3 file (.gff3)	Figshare (https://doi.org/10.6084/m9.figshare.21429219.v3) [7]
Data set 24	*Petrea volubilis* Working Gene Models Proteins	fasta file (.fa)	Figshare (https://doi.org/10.6084/m9.figshare.21429219.v3) [7]
Data set 25	*Petrea volubilis* Working Gene Models Functional Annotation	text file (.txt)	Figshare (https://doi.org/10.6084/m9.figshare.21429219.v3) [7]

The *P. volubilis* genome was annotated as described previously [29]. In brief, repetitive sequences were identified in the unscaffolded contigs using RepeatModeler (v2.0.1) [30] and protein-coding genes removed from the library using ProtExcluder (v1.2) [31]. The custom repetitive sequences were then added to the Repbase Viridiplantae repeats (v20150807) [32] and used to mask repeats using RepeatMasker (v4.1.0) [30] with the parameters -s -nolow -no_is -gff (Table 1, Data file 8, [7]); 57.8% of the genome was masked. RNA-seq reads from five libraries (Table 1, Data file 1, Data sets 6, 7, 8, 9, & 10, [7, 23–27]) were cleaned with Cutadapt (v2.9) [33] using a quality cutoff of 10 and a minimum length 100 nt and then aligned using HISAT2 (v2.2.0) [34] with a maximum intron length of 5000 bp. Gene predictions were generated with BRAKER2 (v2.1.5) [35] using the RNA-seq alignments as hints. Final gene models were refined using the RNA-seq transcript assemblies using two rounds of PASA2 (v2.4.1) [36, 37] and genome-guided transcript assemblies created from the RNA-seq alignments using Stringtie (v2.1.1) [38]. Gene models were annotated using alignments to the predicted *Arabidopsis thaliana* proteome, Pfam database, and transcript evidence as described previously [29]; a total of 49,169 high confidence models (30,982 genes) within the 56,052 working models (37,610 genes) were annotated (Table 1, Data file 9, [7]). High confidence models within the working model set were defined by either protein evidence (alignment to Arabidopsis or Pfam domain and/or expression evidence (TPM > 0). Representative models, both working and high confidence, were defined as the model for each locus (gene) with the longest CDS. BUSCO assessments (v5.4.3 and embryophyta_odb10) of the annotation revealed 89.9% and 88.5% of BUSCO genes in the working gene model and representative high confidence gene model set, respectively (Table 1, Data file 7, [7]). The final genome annotation was transferred from the scaffolds to the chromosomes using Liftoff (v1.6.3) [39] with the parameters -a 0.9 -s 0.95 -exclude_partial -cds -polish.

## Limitations

*Petrea volubilis* is heterozygous and we purged haplotigs in the assembly process. This likely contributed to the reduced N50 contig size (0.53 Mb) and the slightly larger assembly size (480.2 Mb) compared to the estimated genome size from flow cytometry (445 Mb). However, based on BUSCO scores, a mere 4.3% of the orthologs were duplicated in the assembly suggestive that we removed the majority of alternative haplotigs. Future efforts using near-perfect long genomic reads such as PacBio HiFi or Oxford Nanopore Technologies Q20 + platforms would permit a haplotype-resolved genome assembly.

## Supplementary Information

**Additional file 1: Data file 1.** *Petrea volubilis* libraries used in this study. **Data file 2.** Genomescope k-mer frequency distribution plot. **Data file 3.** Hi-C contact map. **Data file 4.** Assembly metrics for the *Petrea volubilis* assembly. **Data file 5.** Pseudomolecule lengths and gap content for the *Petrea volubulis* assembly. **Data file 6.** KAT k-mer comparison plot. **Data file 7.** Benchmarking universal single copy orthologs (BUSCO) results on the *Petrea volubilis* assembly and annotation. **Data file 8.** Repetitive sequence content in the *Petrea volubilis* assembly. **Data file 9.** *Petrea volubilis* gene annotation summary.

## Data Availability

All raw sequence data is available in the National Center for Biotechnology Information under BioProject ID PRJNA534065 (https://identifiers.org/bioproject:PRJNA534065;[11, 15, 16, 21–27]). The assembled genome is available in Genbank under the accession JAOWBU000000000 (https://identifiers.org/assembly:GCA_026212405.1; [28]) and in Figshare (https://doi.org/10.6084/m9.figshare.21429219.v3, [7]). A summary of data sets is available in Table 1 and are available on Figshare (https://doi.org/10.6084/m9.figshare.21429219.v3, [7]).

## Abbreviations

BUSCO: Benchmarking Universal Single Copy Orthologs
PacBio: Pacific BioSciences
